# Supplementary material for: Stochastic agent-based modeling of tuberculosis in Canadian Indigenous communities
Source: BMC Public Health. 2017 Jan 13;17:73. doi: 10.1186/s12889-016-3996-7 (PMC5237134; doi:10.1186/s12889-016-3996-7)
Supplement: Additional file 1: — Model technical appendix. (DOCX 497 kb) [file 12889_2016_3996_MOESM1_ESM.docx]

# Supplementary Materials

We developed an agent-based simulation model of *Mycobacterium tuberculosis* transmission in the Kivalliq Region of Nunavut, Canada. This model represents individuals within a simulated environment, and their interactions, movements, decision-making, and related health states. The region, with a total population of 8955 in 2011, encompasses seven communities, ranging in size from 310 to 2320 people [1]. We used an agent-based approach to account for the small population size. This approach allowed us to model the unique household and community structure in this region, and to record the health states and treatment histories of individuals over time. The model was constructed using the AnyLogic software package (http://www.anylogic.com/). Model parameters were region-specific, wherever possible, or derived from the biomedical literature.

## Population and Community Structure

On model initiation, individuals were assigned an age, sex, household, and community. The initial age distribution of the population was based on 2001 Canadian census estimates for the Kivalliq Region [2]. Each individual was assigned to one of seven communities. Community sizes reflected the Kivalliq region: 3 larger communities (containing ~24% of the population each), 2 intermediate-sized communities (containing ~10% of the population each) and 2 small communities (containing ~4% of the population each). Each individual was also assigned to a household. Household size was assumed to be Poisson distributed with a mean of 4 people per household [1,3]. Each household was located within a community. We assumed that new households were added annually, at a rate estimated from historical census data on change in number of private dwellings in the region [1,3]. The total number of households on model initiation was 1890. Sex and age-specific mortality rates were based on Nunavut life tables [4]. Upon death, individuals were removed from the model population.

Women aged 15-44 gave birth at a rate equal to the Nunavut live birth rate [5]. During a woman’s first birth event, there was the option for the mother and infant to relocate to a new household and community, to ensure that there were no unoccupied households in the model (due to death of occupants or addition of new households). If there were no empty households, newly born individuals were added to their mother’s household and community. When there were empty households in the model, mother-infant pairs were reassigned to the new household (and community). Each newly established household was assigned a household size (using the same distribution as upon model initialization), and other individuals from within the community were randomly selected for relocation to this new household. Additionally, periodically throughout the year, empty households within a community were filled by randomly relocating individuals from within a community to these households, until the assigned household size was reached.

## Natural History of Tuberculosis

The TB natural history component of the model (Figure 1) represented each individual’s health state over time and incorporated the following stages: susceptible, latent infection, active disease, and resusceptible. This component of the model was based on a model of TB transmission developed by Abu-Raddad et al. [6]. The model also included an ‘on-treatment’ state for individuals with active disease who had been diagnosed and were receiving treatment. Parameters describing the transitions between states are presented in Table 1. To capture age-related differences in TB infection, progression, and management, we classified individuals aged <15 years as ‘children’, and those aged ≥15 as ‘adult’. Susceptible individuals became infected by a transmission event following contact with an infected member of their social network. We assumed that the majority of transmission of TB occurred within the household. We also included the community network (encompassing all persons living within an individual’s community) to allow for the investigation of the contribution of casual community contacts to TB transmission. Within the community network, we implemented age-assortative mixing: children (<15 years) mixed only with other children, while adults mixed with other adults. Mixing within these groups was assumed to be random. Upon infection, individuals entered the latent infection state. This state could be fast or slow progressing, reflecting the fact that the majority of individuals remain latently infected for a long period of time without progressing to clinically-apparent infection (‘latent slow’), whereas some cases develop clinical disease rapidly (‘latent fast’). Slow progressors could not develop active disease in the first 5 years after infection [7,8]. Upon progression to active disease, individuals could develop pulmonary disease of low or high transmissibility, or extrapulmonary disease. The proportion of individuals entering the high and low transmissibility states were based on data from Kivalliq, with individuals with smear positive TB assigned to the high transmissibility state. Children with pulmonary TB were more likely to enter the low transmissibility state than adults. We assumed that low transmissibility pulmonary TB was less infectious than high transmissibility TB, and that extrapulmonary disease was not infectious to others. Individuals left the active TB state via spontaneous recovery without treatment, diagnosis and treatment, or death.

For simplicity, we assumed all active cases were correctly diagnosed and initiated treatment. Upon initiating treatment, individuals were assumed to no longer be infectious. Time between developing active TB and starting treatment differed depending on disease presentation (assumed to be shorter for highly transmissible TB). Time on treatment was estimated from treatment data for the Kivalliq region, with a proportion of individuals lost to follow-up.

The model included re-infection, and adopted the approach of previous models [6,9] in assuming that successful treatment of or recovery from active or latent TB provided a degree of protection against re-infection.

## Contact Tracing and Latent Tuberculosis Infection Screening

In the absence of data on latent tuberculosis infection (LTBI) screening in Kivalliq, we used estimates for Iqaluit, Nunavut on annual passive screening for TB [10]. We assumed that susceptible, latently infected and undiagnosed individuals with no prior history of treatment for LTBI or active disease could undergo screening (with screened individuals randomly selected from the population). Those diagnosed with LTBI and aged >6 months and <65 years could receive treatment. The treatment completion rate was estimated from the literature [10,11]. Those diagnosed with active disease entered the appropriate active disease under treatment state. Based on Canadian guidelines, previously treated individuals were not subjected to passive screening and were not offered re-treatment for LTBI [11].

Data on contact tracing completion and outcomes for the province of Saskatchewan were used to estimate the baseline timeliness of contact tracing in Kivalliq [12]. Given the model structure (i.e., random transmission within the community), we assumed that only household members of diagnosed index cases were identified for contact tracing. We assumed that household contacts with LTBI (meeting age and treatment history criteria) were offered treatment, with the same proportion as above completing treatment [10,11].

## Model Calibration

We used model calibration to estimate the number of individuals with latent, undiagnosed or previously treated TB upon model initiation, as well as the annual number of respiratory contacts sufficient to transmit infection. In the absence of data on the demographic characteristics of individuals with latent, undiagnosed active, or previously treated TB, we assumed that the initial number of people in each of these states was distributed randomly in the population. In our base case, we assumed that 5% of respiratory contacts sufficient for transmitting TB occurred within the community. To account for model stochasticity and enable comparison between interventions, we assigned a fixed seed and tested 1000 parameter sets (selected from the range presented in Table 1); the best parameter set was determined by minimizing the least squares difference between cumulative model-projected diagnosed pulmonary TB cases and reported pulmonary TB cases for the Kivalliq region between 1999 and 2012. As we were interested in exploring the impact of assumptions around the role of community transmission on TB dynamics in this setting, we repeated the calibration process assuming that 1% or 15% of respiratory contacts occurred within the community. This process was replayed 10 times, to generate a total of 10 best-fit parameter sets for each value of community transmission.

## Supplementary Analyses

Given the variability in projected TB dynamics in the 10-year period following the model calibration period, we conducted additional analyses to assess the robustness of our findings. To do this, we used two approaches.

*Approach 1.*

In the first approach, we applied the interventions during the 14-year period for which we have surveillance data, a period for which we know that there was significant TB activity in the region. In other words, we asked the question: ‘what would have happened to TB transmission in Kivalliq between 1999-2012 if we had been able to apply each of the different proposed interventions?’ The model was calibrated as described above, under the base case assumptions. For each of the best-fit parameter sets, the model was then re-run with each of the proposed interventions implemented. We compared the cumulative number of incident and diagnosed active TB cases projected to occur in the presence of each intervention to the number that occurred in the base case.

Findings were generally consistent with those in our main analysis (Supplementary Figure 1**)**, although the increased housing scenario had a clearer impact on reducing active TB cases.

**Supplementary Figure 1.** **Projected change in TB incidence and diagnoses if different TB control interventions had been applied during the time period 1999-2012.** The midpoint of boxes represents the median percent change in the outcome of interest, relative to the base case, with the upper and lower bounds representing the 25^th^ and 75^th^ percentiles of percent change, respectively and the bars indicating 1.5 times the interquartile range. Results are based on cumulative outcomes over the 14-year time period, assuming 5% respiratory contacts occurring in the community. Intervention details are provided in Table 2**.**

*Approach 2.*

In the second approach, we used a different method to select well-calibrated model realizations and implement interventions. During the calibration process, the parameters describing number of respiratory contacts, as well as initial numbers of individuals with latent, undiagnosed, or previously treated TB were varied within the ranges described in Table 1. For each intervention, we ran ~5,000 simulations and selected (based on mean squared error) a total of 100-200 simulations that best fit the surveillance data describing case detections of pulmonary TB in Kivalliq between 1999 and 2012 (Supplementary Figure 2).

**A**

**B**

Supplementary Figure 2. Model calibration. Model-projected (A) cumulative and (B) annual cases of pulmonary TB (median: solid line; minimum/maximum: dashed lines) compared to surveillance data (circles) for the Kivalliq region of Nunavut. Results are based on ~1200 model realizations, assuming that 5% of respiratory contacts sufficient for transmitting TB occur within the community.

These best-fit simulations were then used for the analysis of each intervention. Intervention impact was measured as cumulative incidence of TB and diagnoses of active cases over a 10-year period. We calculated the mean and 95% confidence intervals. Note that, in contrast to the main analysis, we are not considering the relative impact of the interventions (as we are no longer comparing intervention impact within a specific outbreak trajectory), but instead are presenting absolute numbers of cases. This approach was applied to the 5% and 15% community transmission scenarios, assuming a 10-year time horizon. The absolute numbers of projected cases were approximately 10-fold higher in the 15% community transmission scenario.

For both the 5% and 15% community transmission scenarios, rapid treatment was projected to significantly reduce the number of incident TB infections and diagnosed active cases, relative to the base case (Supplementary Figures 3 and 4). The other interventions evaluated did not have an appreciable impact on TB burden.

**A**

**B**

Supplementary Figure 3. Projected impact of different interventions on TB burden using alternate calibration approach, assuming 5% community transmission. (A) TB incidence and (B) diagnoses of active cases over a 10-year intervention period were evaluated assuming that 5% of respiratory contacts sufficient to transmit infection occurred within the community. Results are based on 200 realizations of each intervention scenario and are presented as mean values with corresponding 95% confidence intervals. Intervention details are provided in Table 2 of the main text.

**A**

**B**

Supplementary Figure 4. Projected impact of different interventions on TB burden using alternate calibration approach, assuming 15% community transmission. (A) TB incidence and (B) diagnoses of active cases over a 10-year intervention period were evaluated assuming that 15% of respiratory contacts sufficient to transmit infection occurred within the community. Results are based on 100 realizations of each intervention scenario and are presented as mean values with corresponding 95% confidence intervals. Intervention details are provided in Table 2 of the main text.

References

1. Statistics Canada. Keewatin, Nunavut (Code 6205) and Nunavut (Code 62) (table) Census Profile. 2011 Census. Stat. Canada Cat. no.98-316-XWE. 2011. http://www12.statcan.ca/census-recensement/2011/dp-pd/prof/index.cfm?Lang=E. Accessed 16 Jul 2014.

2. Statistics Canada. Community Highlights for Keewatin Region. Cat no. 93F0053X. Community Profiles. 2001. http://www12.statcan.ca/english/profil01/CP01/Index.cfm?Lang=E. Accessed 16 Jul 2014.

3. Statistics Canada. Age and sex, 2006 Census. Cat. No. 97-577-XIE. 2007. http://www5.statcan.gc.ca/bsolc/olc-cel/olc-cel?catno=97-551-XWE&lang=eng. Accessed 16 Jul 2014.

4. Statistics Canada. Life Tables, Canada, Provinces and Territories, 2007 to 2009 (84-537-X). 2013. http://www.statcan.gc.ca/pub/84-537-x/2013003/tbl-eng.htm 2013. Accessed 16 Jul 2014.

5. Nunavut Bureau of Statistics. Nunavut live births by age of mother and total fertility rate. 2013. http://www.stats.gov.nu.ca/en/Population births.aspx. Accessed 16 Jul 2014.

6. Abu-Raddad LJ, Sabatelli L, Achterberg JT, Sugimoto JD, Longini IM, Longini Jr. IM, et al. Epidemiological benefits of more-effective tuberculosis vaccines, drugs, and diagnostics. Proc. Natl. Acad. Sci. U. S. A. 2009;106:13980–5.

7. Vynnycky E, Fine PE. The natural history of tuberculosis: the implications of age-dependent risks of disease and the role of reinfection. Epidemiol. Infect. 1997;119:183–201.

8. Horsburgh C. Priorities for the treatment of latent tuberculosis infection in the United States. N. Engl. J. Med. 2004;350:2060–7.

9. Guzzetta G, Ajelli M, Yang Z, Merler S, Furlanello C, Kirschner D. Modeling socio-demography to capture tuberculosis transmission dynamics in a low burden setting. J. Theor. Biol. 2011;289:197–205.

10. Alvarez GG, VanDyk DD, Aaron SD, Cameron DW, Davies N, Stephen N, et al. TAIMA (Stop) TB: The Impact of a Multifaceted TB Awareness and Door-to-Door Campaign in Residential Areas of High Risk for TB in Iqaluit, Nunavut. PLoS One. 2014;9:e100975.

11. Public Health Agency of Canada. Canadian Tuberculosis Standards, 7th Edition: 2013.

12. Tian Y. Agent-based modeling and system dynamics modeling on transmission of tuberculosis in Saskatchewan. 2012. Thesis. University of Saskatchewn.
